# Gastric Crohn’s Disease With Ileocolonic Involvement in a Patient With Chronic Recurrent Multifocal Osteomyelitis: A Rare Case Presentation

**DOI:** 10.7759/cureus.84586

**Published:** 2025-05-21

**Authors:** Abdul Ghaffar, Fraz Ahmad, Joseph Collum, Hamid Mushtaq, Abdulrahman Abouzaid

**Affiliations:** 1 Gastroenterology and Hepatology, East Lancashire Hospitals NHS Trust, Blackburn, GBR

**Keywords:** auto immune, connective tissue disorder, crohn’s disease (cd), gastric crohn's disease, gastroenterology and endoscopy, gastro-intestinal, inflammatory bowel disease, osteo-myelitis, rheum

## Abstract

Gastric Crohn’s disease is a rare manifestation of Crohn’s disease affecting the stomach, unlike its more common forms that primarily affect the ileum and colon. This is a case of an 18-year-old female who was referred to gastroenterology for investigation of iron deficiency anaemia, which was first noted when she was 15 years old. Investigation for persistent iron deficiency anaemia (IDA) revealed high faecal calprotectin and a positive faecal immunochemical test (FIT). Endoscopy was done, which revealed mild haemorrhagic gastritis and a few superficial gastric erosions. Biopsies taken from the gastric fundus indicated ill-defined granulomas with no significant active inflammation and no presence of *Helicobacter pylori*. Colonoscopy done up to the caecum was normal endoscopically. However, the random colonic biopsies taken from the ascending colon revealed mild active chronic inflammation. Granulomas were seen, in keeping with Crohn’s colitis, making this a case of gastric Crohn’s disease with ileocolonic involvement. The patient was treated with budesonide for acute symptom management. Following discussion in the inflammatory bowel disease (IBD) multi-disciplinary team meeting (MDT), she was commenced on Infliximab. Follow-up evaluations showed no significant relapse episodes. This case also highlights the importance of family history and genetic evaluation in patients with potential hereditary or autoinflammatory conditions, which would aid in narrowing the diagnostic spectrum.

## Introduction

Crohn’s disease (CD) is a type of inflammatory bowel disease (IBD) that can impact any area of the gastrointestinal tract, but it most commonly affects the ileum. While the exact cause of this disease is still not well understood, it’s thought to stem from various genetic, environmental and immunological factors [1]. Gastric involvement in CD is uncommon and presents a unique clinical challenge due to its rarity and the nonspecific nature of its symptoms, which often overlap with other gastrointestinal conditions. This report describes an 18-year-old female, known to have chronic recurrent multifocal osteomyelitis (CRMO), who presented with a history of tiredness and fatigue, suspected to be related to iron deficiency anaemia (IDA), and intermittent fresh blood on toilet paper over the past 3-4 years.

CRMO is closely linked to IBD. CRMO has a strong association with inflammatory conditions, including IBD, suggesting a similar pathophysiology that may involve the immune system and genetic susceptibility.

## Case presentation

**Figure 1:**
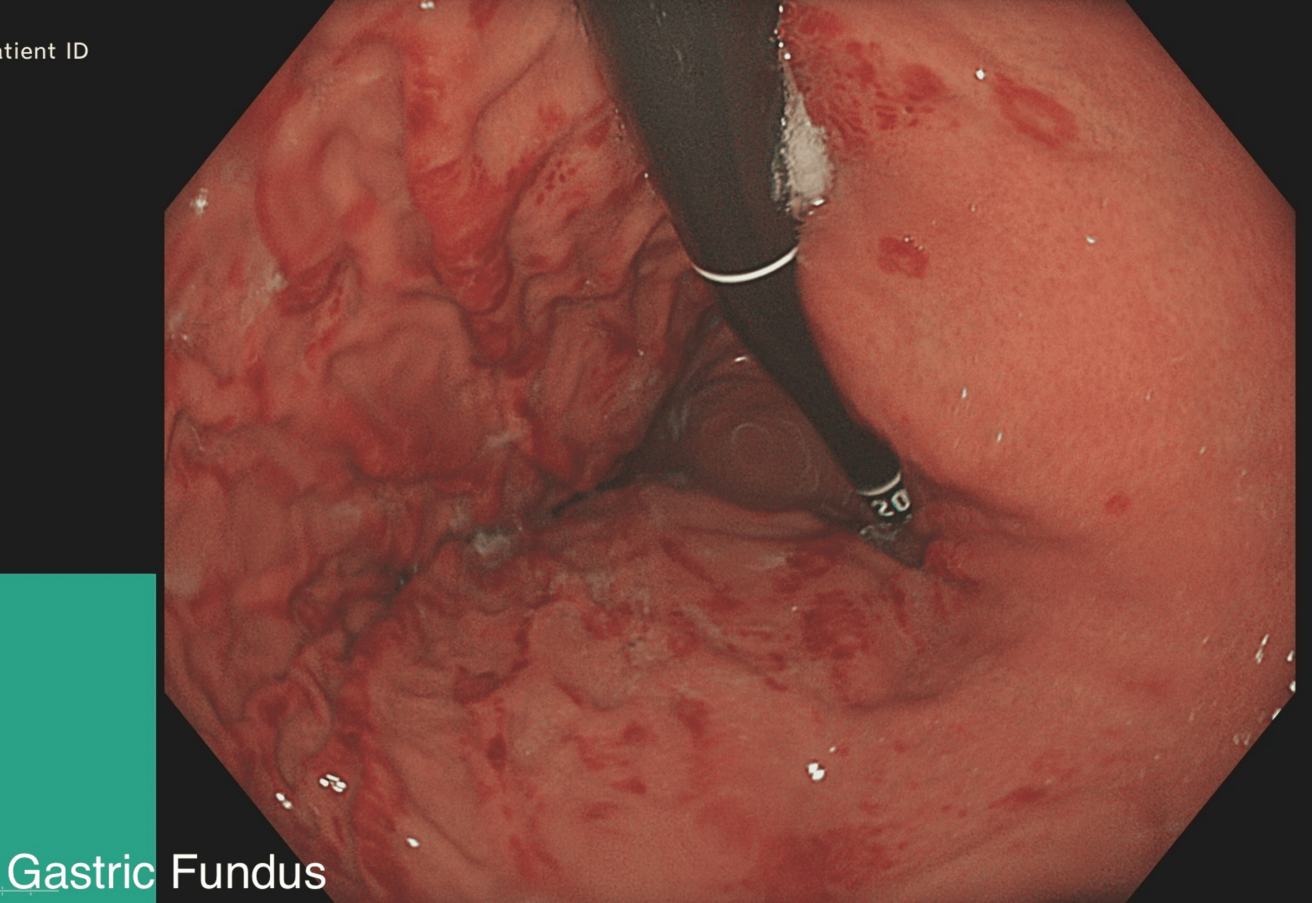
Gastric Fundus: Mild haemorrhagic gastritis and a few superficial gastric erosions.

**Figure 2:**
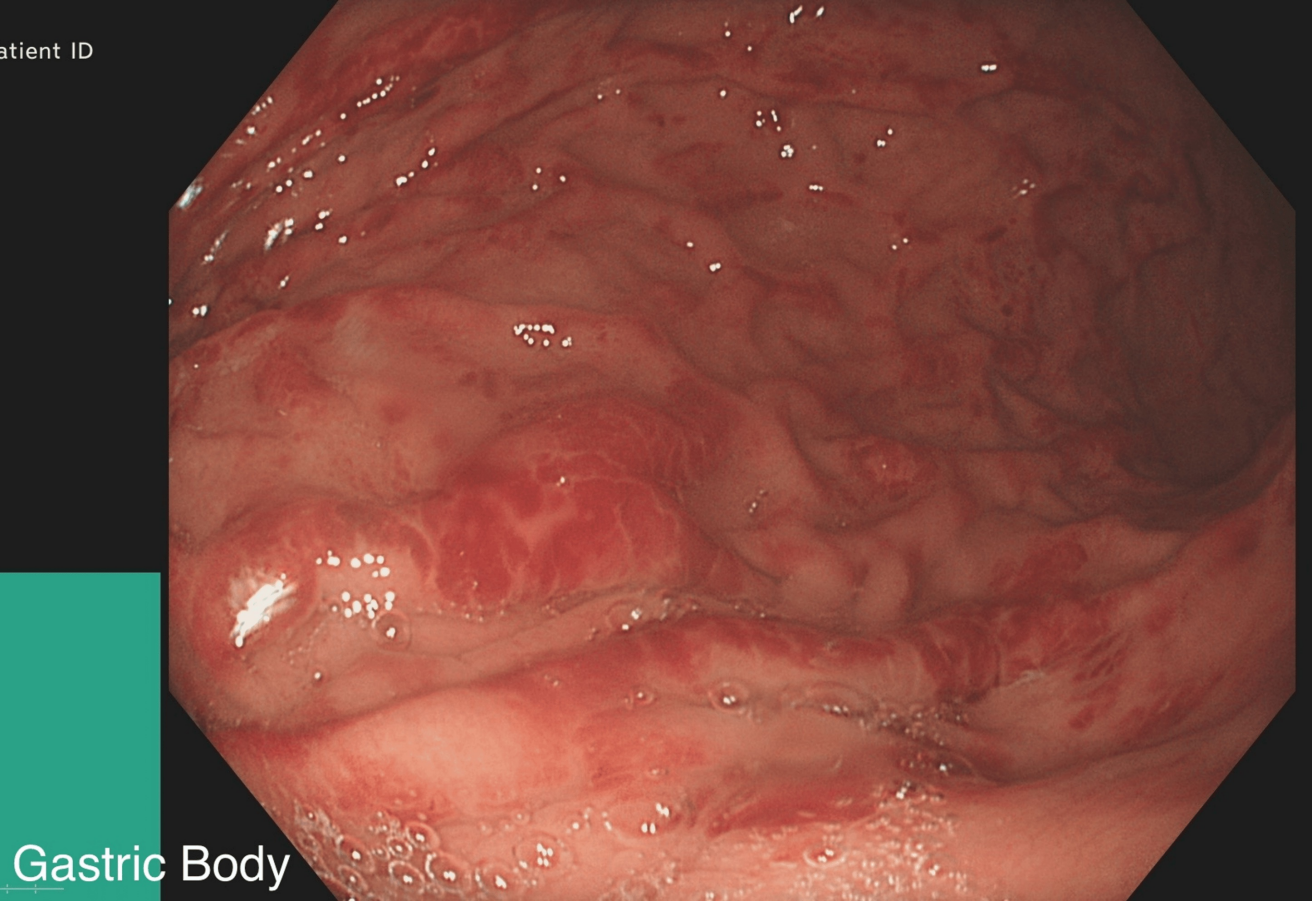
Gastric Body: Mild haemorrhagic gastritis and a few superficial gastric erosions.

**Figure 3:**
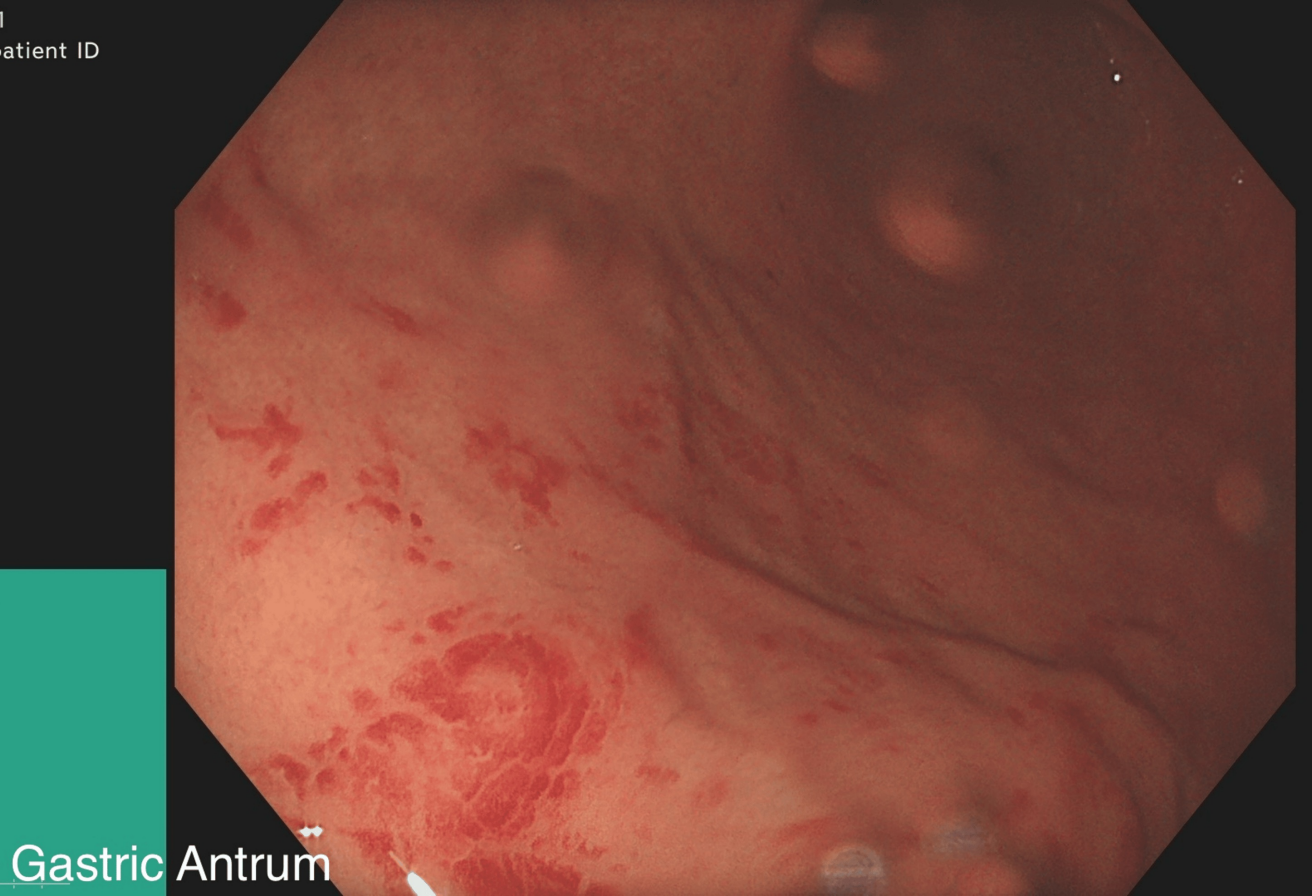
Gastric Antrum: Mild haemorrhagic gastritis

An 18-year-old female, referred to the gastroenterology department for investigation of iron deficiency anaemia (IDA), first noted when she was 15 years old. She was under the care of a Rheumatologist for CRMO (genetically related cause), found to have anaemia and hence referred for further investigations. Investigation for persistent IDA revealed low iron levels, negative Celiac screen and high faecal calprotectin. In terms of her symptoms, she mentioned opening her bowels once every 3 days with occasional fresh blood for the last 3 years and occasional abdominal pain. The physical examination was normal. Owing to the patient’s persistent symptoms, an upper endoscopy was performed, which revealed mild haemorrhagic gastritis and a few superficial gastric erosions (Figures 1-3).

Biopsies taken from the gastric fundus indicated ill-defined granulomas with no significant active inflammation and no presence of *H. pylori*. This was consistent with gastric CD. Biopsies from the D1 and D2 regions were unremarkable.

**Figure 4:**
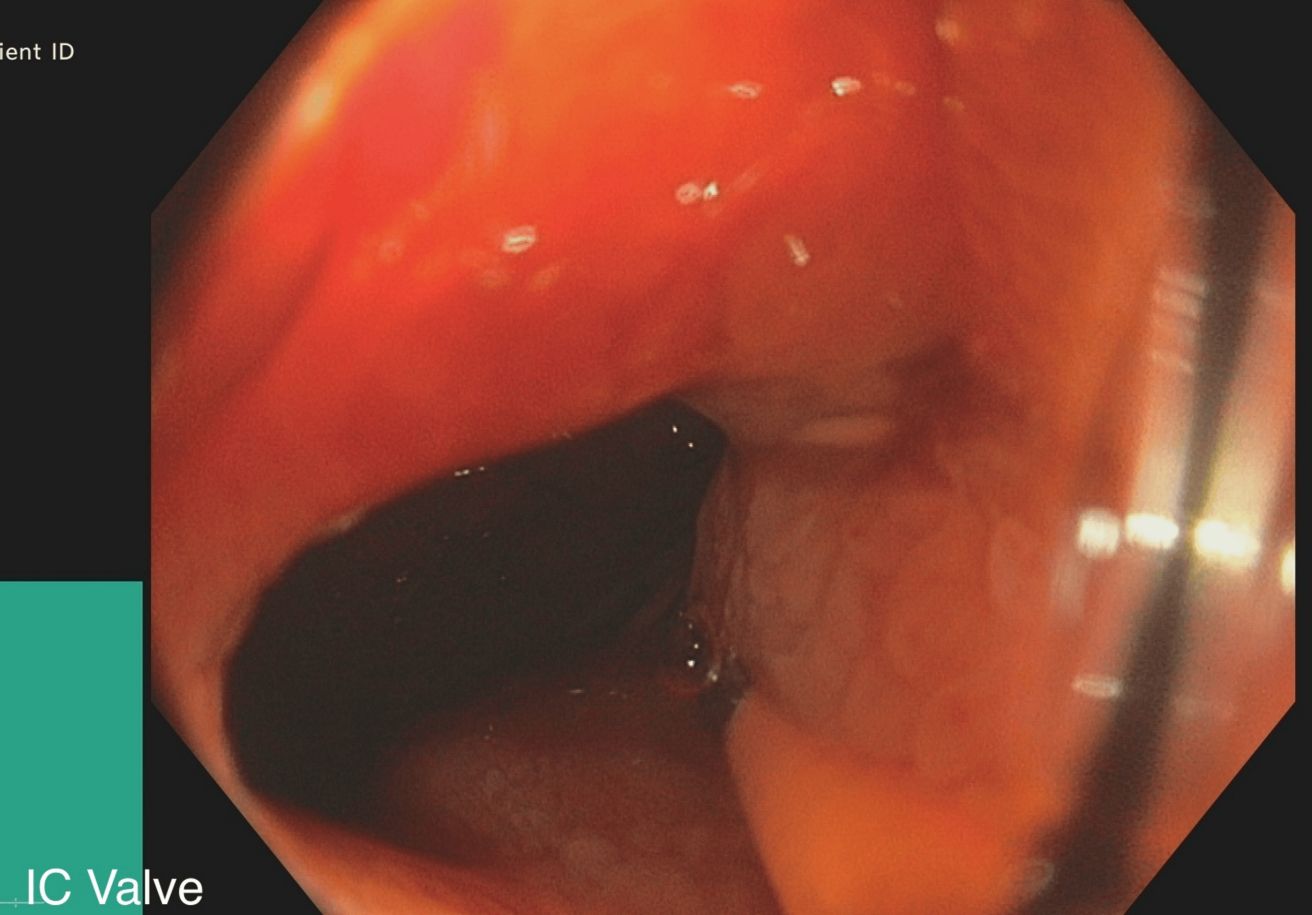
Ileocecal Valve: Endoscopically, no significant abnormalities.

Colonoscopy was performed up to the ileocecal valve (Figure 4), and biopsies were taken from the ileum. No haemorrhoids were found during the colonoscopy. The mucosa from the proximal ascending colon to the rectum appeared normal. Biopsies from the terminal ileum showed active inflammation with areas of ulceration but no granulomas. Biopsies from the proximal ascending colon indicated mild active chronic inflammation in the lamina propria, with foci of cryptitis. Few granulomas were seen. The descending colon showed no significant inflammation, but granulomas were again observed. Rectal mucosa and biopsies were normal. Findings were consistent with Crohn’s colitis. MRI of the small bowel revealed abnormal thickening and ulceration of the terminal ileum spanning 15 cm, compatible with CD. No abscesses or fistulas were observed.

The above findings were consistent with gastric CD with ileocolonic involvement.

The patient was prescribed an 8-week course of budesonide followed by a tapering regimen of steroids. She was strongly advised to discontinue non-steroidal anti-inflammatory drugs (NSAIDs) immediately and was referred to the IBD multi-disciplinary team meeting (MDT). After completing the pre-biologic evaluation, she started treatment with Infliximab. Dietitians were also involved as part of the overall treatment plan for her IBD. The patient’s disease activity was monitored during several follow-up appointments, and there were no significant relapse episodes.

## Discussion

In most cases, diagnosing CD relies on a mix of common clinical signs, lab results, endoscopy, and histopathological findings. However, it becomes challenging to diagnose when the disease presents atypically, like when there’s gastric involvement in CD.

Gastric CD can present with a mix of non-specific symptoms such as nausea, vomiting, bloating, weight loss and postprandial abdominal discomfort. These symptoms may present in other gastric aetiologies such as NSAID-induced gastritis, *H. pylori*, Tuberculosis, Ménètrier’s disease, gastrinoma, collagen vascular disease and lymphoma [2]. These conditions are important to rule out to establish a diagnosis of gastric CD.

Findings of upper gastrointestinal endoscopy for gastric CD can appear grossly normal or show a mix of signs like oedema, erythema, ulcers, nodularity and a cobblestone-like appearance. Typically, the antrum is the part that gets affected the most in gastric CD. After ruling out another source of disease, linking these findings from an upper endoscopy, along with histological results and serological testing, assists in diagnosing gastric CD [2].

In this case, the ileum and descending colon were also involved, which was confirmed on biopsy, and henceforth the diagnosis of gastric CD with ileocolonic involvement was made.

In the context of management, therapy aims to control inflammation, relieve symptoms and prevent complications. To manage acute exacerbations and severe symptoms, prednisone 40 mg once daily is often used as an induction therapy. Immunomodulators like Azathioprine, Anti-TNF therapy, Ustekinumab and Vedolizumab are used for long-term maintenance therapy [3]. Regular follow-up with endoscopic evaluation is necessary to monitor disease activity, adjust treatment as needed and prevent complications such as gastric outlet obstruction, which is reported in a few cases [4].

This patient was also known case of CRMO, which was established to be due to a genetic cause. One of her siblings also suffered from Multifocal Osteomyelitis. CRMO is a rare disease that usually presents in children and adolescents. It is characterized by aseptic inflammation of long bones, in particular the metaphyses are mostly affected, although the patient can have lesions anywhere in the skeleton [5].

While CRMO is considered rare, it is increasingly recognized in clinical practice now. CRMO is known to be associated with IBD, however, there are only a few documented case reports of children having CRMO along with IBD, making it a rare but important association. Previous studies have revealed that in the majority of patients, the symptoms for CRMO manifest earlier than the symptoms for IBD, typically predating IBD between 3 months to 7 years [6]. This was the case in our patient as well, who had prior symptoms and a diagnosis for CRMO.

CRMO is linked to several inflammatory conditions, including psoriasis, palmoplantar pustulosis, Sweet's syndrome, Takayasu's arteritis, Wegener's granulomatosis, spondyloarthropathy, pyoderma gangrenosum, and IBD [7]. Kahn was the first to bring up the link between CRMO and IBD. In an unpublished letter, he noted that out of 30 CRMO patients, 5 also had IBD. Particularly, he reported that 4 of them had CD and 1 had Ulcerative colitis [8].

The cause of CRMO isn’t fully understood, but its strong link to inflammatory conditions hints at a similar pathophysiology, possibly involving the immune system with an aspect of genetic susceptibility. Previous studies indicate that bone inflammation in CRMO patients is due to an irregular immune response targeting the bones. Similarly, one of the main factors in IBD is a dysregulated immune response. Multiple theories have been put forward, with one highlighting that cytokines released from the inflamed gut, especially interleukin 1, interleukin 6, and tumour necrosis factor-α, could trigger bone inflammation resulting in an extra-intestinal manifestation of IBD [7,9].

Recent research in mouse models of Chronic multifocal osteomyelitis (CMO) indicates that IL-1 is a key player in the disease, and also that certain dietary changes can influence the CMO microbiome, potentially stopping osteomyelitis from developing [10]. It is established that interleukin 1β (IL-1β) is a significant mediator of inflammation and tissue damage in IBD [11].

Further exploration into the interrelation between IBD and CRMO would be beneficial in identifying interlinked cytokine-mediated pathways and hence formulating management with targeted biologic therapies. It’s important to note that studies have shown that using immunosuppressive drugs to treat bowel disease led to a reduction in bone inflammation for patients of CRMO with IBD [9].

Most of our understanding of gastric CD comes from small case studies, highlighting the need for more extensive research to provide a greater understanding of this uncommon manifestation of CD. Future studies are needed for identifying predictive markers for gastric CD, examining the interrelation between CD and other connective tissue disorders, improving treatment methods, and investigating long-term outcomes and responses to therapies.

## Conclusions

This case not only highlights the diagnostic challenges and complexities while dealing with gastric CD but is also thought provoking in the context of associating rare auto inflammatory disorders like CRMO and IBD. The patient initially presented with symptoms of IDA and was referred by the Rheumatology team for further evaluation. Endoscopy and colonoscopy revealed mild haemorrhagic gastritis and a few superficial gastric erosions. While these are non-specific findings, in conjunction with histological analysis, they served as supportive evidence for a diagnosis of gastric CD with Ileocolonic involvement.

Treatment with budesonide for acute symptom management followed by infliximab for long-term maintenance was initiated, and the patient’s disease activity was closely monitored through regular follow-ups. This case highlights the importance of considering IBD as a possible diagnosis in young patients who already have other autoinflammatory disorders, presenting with IDA and abdominal symptoms. The prognosis for gastric CD is unclear, and continuing monitoring is crucial to adjust treatment and prevent complications. This case highlights the need for further research to deepen our understanding of gastric CD and emphasizes the importance of exploring shared pathophysiology between IBD and other autoinflammatory disorders, such as CRMO.
